# Misconceptions Yield Misleading Results. Comment on Wong et al. Comparative Study of Eclipse and RayStation Multi-Criteria Optimization-Based Prostate Radiotherapy Treatment Planning Quality. *Diagnostics* 2024, *14*, 465

**DOI:** 10.3390/diagnostics15121493

**Published:** 2025-06-12

**Authors:** Björn Hårdemark

**Affiliations:** RaySearch Laboratories AB, SE 104 30 Stockholm, Sweden; bjorn.hardemark@raysearchlabs.com

## 1. Introduction

The study by Wong et al. [1] presents a comparison of multi-criteria optimization (MCO) for prostate radiotherapy planning in RayStation and Eclipse. However, the methodology described in their article does not provide sufficient detail on how either software was configured, preventing readers from properly assessing the validity of the authors’ conclusions. After direct correspondence with the authors, they supplied a detailed description of how RayStation was configured, and it then became clear that their findings were based on incorrect usage of the system. This issue is critical because it significantly undermines the validity of their conclusions regarding RayStation’s performance.

## 2. Incorrect Usage of Objectives

In their article, Wong et al. state that they aimed to minimize rectum D50 (or V50), but they do not specify how this was implemented within RayStation. From our correspondence with the authors, we learned that they did not properly define the optimization objective. Instead of instructing the system to minimize rectum dose as much as possible, they set the system to minimize only down to 50 Gy. This effectively placed an artificial lower bound on dose reduction, preventing the optimizer from achieving optimal rectal sparing. While this kind of threshold can be useful in specific scenarios, such as structures overlapping the target or dealing with multiple dose levels, it was misapplied in this case, leading to suboptimal plans.

The other trade-off objective was to achieve a minimum of 76 Gy to 98% of the target. This would have been a reasonable objective if not for the fact that the authors also introduced a constraint requiring a minimum of 76 Gy to 99% of the target. The trade-off objective for the PTV was therefore irrelevant, which the system also noted in an error message when generating the Pareto plan, and the generation was typically interrupted before the requested number of plans had been produced.

Due to the above problems, the configuration used by Wong did not correctly translate their intention to the RayStation system, and the system consequently did not minimize the rectum D50 (or V50). In correspondence, the authors pointed out that they had followed steps from the RayStation user manual and that their usage was, in this sense, correct. However, merely following the manual is not sufficient to guarantee that your planning intentions are correctly realized in the system.

## 3. Correcting the Optimization Approach

Following the guidelines of Craft [2], we modified the rectum dose trade-off objective to 0 Gy. We used an equivalent uniform dose (EUD) function (with a = 2) instead of a dose–volume-based criterion, as it encourages dose reduction across the entire rectum. Furthermore, we modified the PTV trade-off objective to achieve a minimum of 76 Gy to 100% of the PTV rather than 98%. While this is still a near duplicate of the constraint, it is at least a mathematically sound combination. We also introduced a maximum dose constraint to the entire patient volume at 107% of 76 Gy to ensure that our method did not produce plans that would not be clinically feasible.

## 4. Comparison Setup

We could not access the patient cases used by Wong et al., so to perform a comparison, we used 25 other prostate cancer cases from another institution. The datasets were collected in accordance with all applicable institutional and ethical guidelines and were provided as fully anonymized data to RaySearch for research and development purposes. The median PTV volume was 140 cm^3^ (somewhat larger than the median of 98 cm^3^ in the cases used by Wong et al.). Secondary target volumes with lower dose levels were disregarded. We used the same software version (RayStation 12A, RaySearch Laboratories, Stockholm, Sweden) and dose engine (Collapsed Cone). We replicated the Wong configuration as it was described in our correspondence with the authors and made plans for all patients as a basis for comparison. We refer to these as the “Wong” plans. New plans were then made with our above-mentioned corrections, and we refer to these as the “Craft” plans.

To make sure that results were not unnecessarily impacted by partial convergence or dose approximations, we allowed 100 iterations per Pareto plan and ran two times 100 iterations for the deliverable plans. We also allowed the system to create 20 Pareto plans for each case. These settings were applied equally to the Wong and Craft plans.

Wong et al. used the navigation sliders in RayStation to manually produce five different navigated states per plan. However, since there was no actual trade-off, the plans did not vary meaningfully. In our study, we used the built-in feature of automatic navigation to obtain the plan that best fulfilled the clinical goals (at least 76 Gy to 98% of the PTV and the lowest possible D50 to the rectum).

This study utilized Python scripting in RayStation. Script snippets for the different steps were recorded and put together into a script that could create the plan according to the Wong and Craft configurations, calculate the Pareto plans, auto-navigate to a solution maximizing clinical goal fulfillment, calculate the deliverable plans and related statistics. The processing time per case was approximately 15 min.

## 5. Results

In all cases, the PTV coverage at the 98% level was very close to the prescribed 76 Gy, but the rectum D50 was drastically reduced by our corrections. It is noteworthy that many Wong plans achieved rectum D50 doses below the 50 Gy that was requested. This results from the natural fall-off of the dose away from the PTV, rather than from intentional dose reduction. The D50 doses are then reduced somewhat in the deliverable plans compared to the navigated plans since the system aims to stay below the entire DVH of the navigated plans. For the Craft plans, the navigated and deliverable plans are quite similar, which is the expected behavior of the system.

**Figure 1:**
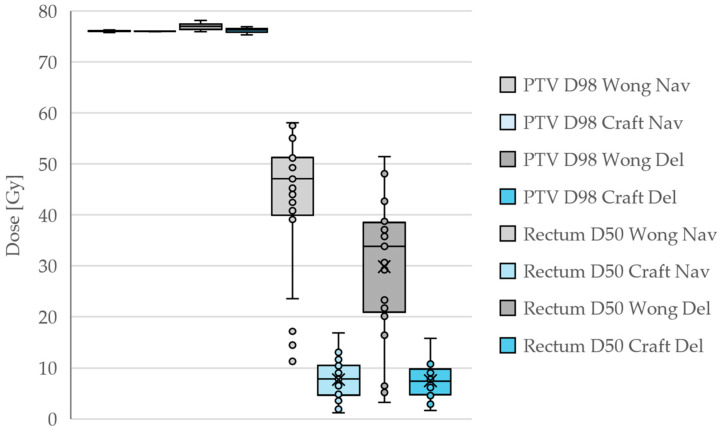
Dosimetric impact of incorrectly (Wong) and correctly (Craft) configured RayStation MCO plans shown for both the navigated plans (Nav) and deliverable plans (Dev). Target coverage is equivalent, but the rectum doses are drastically improved in the Craft plans.

A summary of the PTV D98 and rectum D50 dose statistics for the different plans is shown in Table 1 and Figure 1.

## 6. Conclusions

The conclusions drawn by Wong et al. are a direct consequence of their incorrect optimization setup, not representative of how RayStation would be used clinically. With appropriate adjustments, significantly improved plans were generated. In this study, we have not investigated whether Eclipse was also used incorrectly. Furthermore, since we used different patient data, a direct comparison between our RayStation results and the Eclipse results of Wong et al. is not possible.

A previous study [3] has demonstrated that MCO in RayStation allows novice planners to perform at least as well as experienced planners using conventional optimization, while at the same time being more efficient. Nevertheless, it is crucial that the planner has a basic understanding of what the various functions mean and how their input is interpreted by the system. Wong et al.’s results are an illustration of the impact that a lack of such understanding can have, and in this way, their study provides an important cautionary lesson. However, they do not provide a meaningful assessment of the actual capabilities of RayStation and risk misleading the clinical community.

## Figures and Tables

**Table 1:** Dosimetric impact of incorrectly (Wong) and correctly (Craft) configured RayStation MCO plans. Target coverage is equivalent, but the rectum doses are drastically improved in the Craft plans. Dose levels are presented as averages and 95% confidence intervals.

Structure	Parameter	Wong [Gy]	Craft [Gy]
PTV	D98 (Navigated)	76.18 ± 0.13	76.06 ± 0.03
PTV	D98 (Deliverable)	76.99 ± 0.25	76.25 ± 0.16
Rectum	D50 (Navigated)	42.59 ± 5.29	7.68 ± 1.57
Rectum	D50 (Deliverable)	29.87 ± 5.71	7.50 ± 1.33

## Abbreviations

The following abbreviations are used in this manuscript:

MCO	Multi-criteria optimization
EUD	Equivalent uniform dose
PTV	Planning target volume
